# Optimizing Agility and Athletic Proficiency in Badminton Athletes Through Plyometric Training: A Review

**DOI:** 10.7759/cureus.52596

**Published:** 2024-01-19

**Authors:** Saylee S Shedge, Swapnil U Ramteke, Pratik R Jaiswal

**Affiliations:** 1 Sports Physiotherapy, Ravi Nair Physiotherapy College, Datta Meghe Institute of Higher Education & Research, Wardha, IND

**Keywords:** safety considerations, athletic performance, agility, badminton players, plyometric training

## Abstract

This review study investigates the advantages of plyometric training in badminton, concentrating on the effects on agility, power, speed, and overall athletic performance. It looks at the research on plyometric training in badminton, including training methods, performance effects, and potential injury prevention. The study underlines the value of plyometric training in enhancing agility, letting athletes move swiftly around the court, and producing the explosive force needed for quick and accurate strokes. It also looks at how plyometric training affects jumping ability and explosiveness, with a concentration on vertical jumps and hard smashes. In addition, the research explores the function of plyometrics in injury prevention in the physically demanding sport of badminton. Plyometric training has emerged as a key component in lowering injury risk and extending player life by strengthening muscles and connective tissues and boosting stability. The review study objectively reviews the existing corpus of research to provide a full picture, considering prior studies' strengths and limitations. It gives coaches, athletes, and trainers concrete ideas for incorporating plyometric activity into their badminton training routines. The study gives readers actionable recommendations for improving agility and athletic performance in badminton athletes by tailoring plyometric exercises to the unique demands of the sport and addressing safety concerns. In a sport where margins of victory are frequently razor-thin, embracing the possibilities of plyometric training can provide a competitive edge, ultimately improving the performance and success of badminton competitors.

## Introduction and background

Badminton is a highly competitive racquet sport that requires a unique set of skills such as lightning-fast reflexes, agility, stamina, and explosive strength. Athletes and coaches are continuously looking for new techniques to improve the agility and athletic ability of badminton players in order to improve their performance. Plyometric training, a discipline that stresses explosive muscle contractions and dynamic movements, has emerged as a possible game changer in the endeavour to develop these traits. The importance of agility in badminton cannot be overemphasized. Players must be able to move swiftly across the court, adjust between offensive and defensive roles, and return shots from a variety of angles. The ability to quickly reverse direction, stop momentum on a dime, and maintain balance are all required for success in the sport. For a competitive advantage in the sport's unrelenting tempo and explosive force demands, speed and muscular strength are essential. Plyometric training fits both of these criteria, providing a way to enhance the power and speed required of a well-rounded badminton player. In badminton, jumping ability and explosiveness, as well as agility and power, play a crucial role, allowing players to do gravity-defying leaps to return high shots and execute blistering smashes. In this aspect, plyometric workouts that emphasise vertical jumps and quick contractions have the potential to be game-changers [1].

Plyometric exercises, in addition to enhancing performance, have other benefits. Injury prevention is crucial in the demanding terrain of competitive sports, and badminton is no exception. By strengthening muscles and connective tissues, plyometric training enhances stability and resilience, minimizing the incidence of common badminton injuries. Through an in-depth examination of relevant studies, this paper critically examines the strengths and limitations of plyometric training in the context of badminton. It offers practical suggestions to coaches, athletes, and trainers on how to include plyometrics in their training routines [2]. This review paper lays the groundwork for harnessing the full potential of plyometric training to optimize agility and athletic proficiency in badminton, thereby elevating badminton athletes' performance and competitive advantage as the shuttlecock takes flight and athletes take to the court in pursuit of victory.

The value of agility and athleticism in badminton cannot be overstated. These qualities are critical for success in this fast-paced, precision-driven sport. In badminton, the ability to quickly change direction, pivot, and accelerate in response to the shuttlecock's erratic path is critical [3]. The ability to glide effortlessly and precisely over the court lets players return shots from various angles and successfully cover the floor. In addition to quickness, precision and endurance are vital in badminton. Precision dictates where each shot lands, determining the difference between a winning point and a missed opportunity. Endurance, both cardiovascular and muscular, is required to maintain peak performance during extended rallies and games [4]. Potteiger et al. examined muscle power output and fibre characteristics after an eight-week plyometric and aerobic exercise programme for male subjects. Results showed significant increases in peak power output and fibre area, suggesting muscle fibre size may be a factor [5]. The pursuit of agility and athletic proficiency is critical to the progress of badminton as a competitive sport, giving players a competitive advantage by allowing them to move beautifully, hit forcefully, and respond fast, thus ensuring success on the court [6].

Purpose and scope of the study

This review paper investigates the effect of plyometric training on the agility and athletic proficiency of badminton athletes. Plyometrics, a training method centred on rapid muscle contractions and explosive movements, has the potential to develop key badminton characteristics such as agility, power, speed, and jumping ability [7]. The study objectively reviews the strengths and limits of current studies, shedding light on the efficacy of plyometric training for badminton players. It also focuses on practical applications, including advice to coaches, athletes, and trainers on developing efficient plyometric training routines adapted to specific badminton needs, as well as safety considerations and injury prevention methods. The purpose of this review is to bridge the gap between academic understanding and practical implementation, allowing individuals to reap the benefits of plyometric training in the competitive sport of badminton [8]. Badminton players can acquire a considerable competitive advantage by improving their agility and physical proficiency, hence improving their performance and success [9].

Practical applications

Plyometric training has many practical applications for badminton athletes and has the potential to increase performance significantly. Coaches, athletes, and trainers can employ plyometrics to increase agility, power, speed, and jumping ability. Agility-focused practices such as ladder drills and shuttle runs can help a player's court movement and reactivity [10]. Power and speed workouts, like deep leaps and bounding, help to improve smashes and court coverage. Plyometrics also helps athletes enhance their jumping abilities, which allows them to hit high shots and deliver powerful smashes [11]. Incorporating plyometric exercises into warm-up routines may also help players prepare for explosive actions on the court. However, it is vital to tailor plyometric routines to the specific goals and fitness levels of each athlete, to ensure proper technique, and to address injury prevention [12]. In this respect, plyometric training is a dynamic tool for badminton athletes, helping them to hone their talents and acquire a competitive advantage in the highly competitive world of badminton [13].

## Review

Methodology

**Figure 1:**
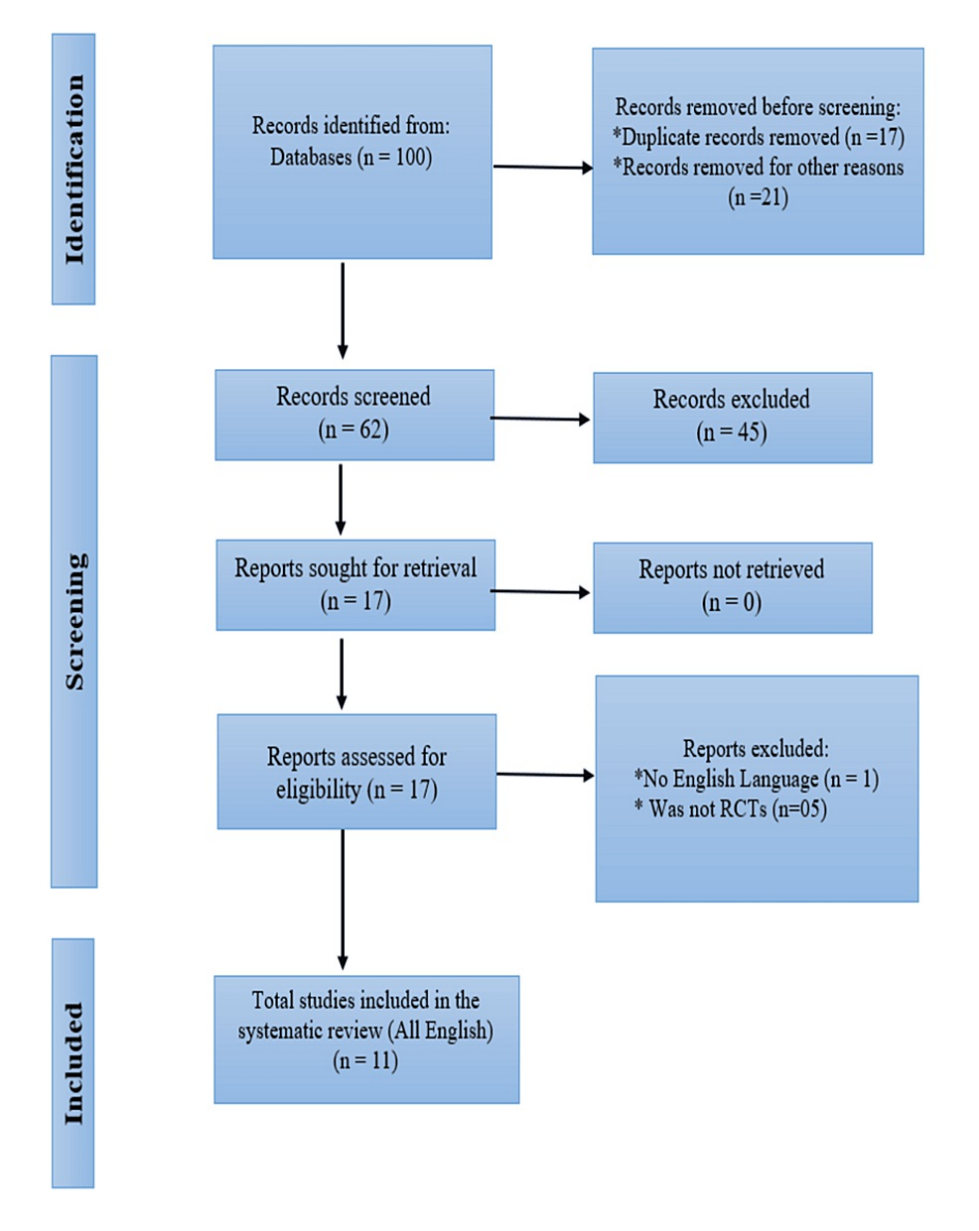
Preferred Reporting Items for Systematic Reviews and Meta-Analyses (PRISMA) diagram of the literature search used RCTs: Randomized controlled trials

While researching the aforementioned topic, a complete and extensive technique was used. The findings were culled from research sources such as PubMed, Google Scholar, Medline (Medical Literature Analysis and Retrieval System Online), Embase (Excerpta Medica Database), and others and exhaustively examined using keywords such as 'Plyometric Training,' 'Badminton Players,' and 'Agility.' Articles written in languages other than English were filtered out and not considered (Figure 1). The editor's note was likewise removed.

Review of literature

From 2014 to the present, articles have been included for the review of literature. Some of the articles summarized in the review are listed in Table 1.

**Table 1:** Summary of the included studies VO2max: Maximal oxygen consumption; VJH: Vertical jump height; SAQ: Speed, agility, quickness

Sr. No.	Study	Participants	Interventions	Outcomes measures	Results
1	Chandra et al. (2023) [8]	102 recreational badminton players were involved in the research.	Experimental group:- Plyometric Training 2 times a week with 2 days rest between sessions; Control group:- Regular training for 3 weeks	Agility- agility T-test, Speed- 30-meter sprint test, Anaerobic Capacity:- broad jump	According to the authors, a unique three-week plyometric training regimen consisting of low- and moderate-intensity activities resulted in considerable increases in agility and speed performance for badminton players. Even minor differences in standing broad jump distances and agility times can have a substantial impact on a match's outcome if a longer regimen is followed.
2	Nugroho et al. (2022) [14]	There were 24 athletes involved in the study.	Plyometric drills and hurdle drills.	Agility- T-drill test, VO2max- multistage fitness test	The study found that the effects of plyometric ladder drills and hurdle drill exercises on badminton athletes' agility differ significantly.
3	Panda et al. (2022) [15]	120 state-level badminton players were included in the study.	Plyometric training:- 2 times/week for 4 weeks, Electromyostimulation training:- 4 times/week for 4 weeks; Control group:- Routine badminton practice + related training.	Agility T-test, 30-m sprint test, standing broad jump, vertical jump height test.	The study discovered that combining four weeks of plyometric training with four weeks of electromyostimulation training increases the sprinting ability and agility of badminton players more than electromyostimulation training alone. When paired with normal sports training, both modalities of training greatly improve VJH performance in badminton players.
4	Khatoon and Thiyagarajan (2021) [16]	34 subjects were included in the analysis.	Group A:- Pilates group - core strength training + regular training; Group B :- Plyometric group - plyometric drills + regular training; Duration for both the groups:- 2 times per week for 6 weeks	Balance- star excursion test, Agility- Illinois agility test, core muscle endurance test	The study looked at how core strengthening and plyometric exercise improved the dynamic balance and agility of elite Indian badminton players. The findings revealed that both groups were useful; however, no treatment program outperformed the other statistically. Future research should investigate how they impact performance and injury prevention.
5	Karatnyk et al. (2021) [17]	30 athletes were recruited and divided into 3 groups	Module 1-Speed, Module 2- Strength, Module 3- Jumping Exercises; Combination 1:- 1-8 weeks – Module 1; 9-16 weeks – Module 2; 17-24 weeks – Module 3. Combination 2:- 1-8 weeks – Module 2; 9-16 weeks – Module 3; 17-24 weeks – Module 1. Combination 3:- 1-8 weeks – Module 3; 9-16 weeks – Module 1; 17-24 weeks – Module 2.	Speed and strength abilities	Prior work in this field may be complemented by the researchers' findings: the deployment of module combinations and individual modules is beneficial in developing badminton players' physical skills. The findings suggest that the training routine for players aged 15 to 17 should emphasize speed and strength. The most successful module combinations are combinations 1 (speed + strength + jumping exercises) and 3 (jumping exercises + speed + strength).
6	Yuksel and Aydos (2019) [18]	101 subjects were included in the study.	Badminton footwork training group; Classic badminton training group; Control group; Duration:- 60 minutes per day and 4 days per week for 16 weeks.	Leg and back strength- dynamometer 30 seconds sit-up test, vertical jump test, anaerobic power measurement, side-to-side agility test, and 4-corner agility test	The study found that children aged eight to 10 benefitted from frequent badminton training in terms of strength and agility performance metrics, with substantial improvements in vertical jump, anaerobic power, back strength, and other agility parameters. In terms of anaerobic power and leg strength, boys outperformed girls. Footwork badminton training exercises are considered superior to classical badminton because of their ability to perform techniques at the appropriate time.
7	Pooja (2019) [19]	There were 30 recreational badminton players involved in the research.	Group A:- plyometric drills; Group B:- core strength training; Duration:- both the groups received 6 weeks of intervention.	Agility- shuttle run test and Illinois agility test, Balance- Y balance test	The researchers investigated the effects of plyometric and core stability workouts on agility and balance in recreational badminton players. Core stability improved agility and balance more than plyometric exercise, hinting that it could be used in injury prevention therapy.
8	Kanagasabai et al. (2017) [20]	30 badminton players were included.	SAQ drills + plyometric training Group and Control group; Duration:- SAQ drills + plyometric training group- 3 alternative days in a week for 8 weeks with 3 sets/exercise/session at 60 to 80% with a progressive increase in load with the number of weeks	Breathing hold time- stopwatch, Vital capacity- wet spirometer	Breathing hold time and vital capacity were improved with SAQ drills and plyometric training. There was a statistically significant difference between the SAQ and Plyometric training groups as compared to the control group.
9	Ozmen and Aydogmus (2017) [21]	The study involved 20 adolescent badminton players.	Plyometric group:- plyometric training + routine training; Control group:- routine exercises; Duration:- both the groups performed the exercises twice a week for a 6-week period	Agility- Illinois agility test, Vertical jump- squat jump test using a contact mat.	Plyometric training increased squat jump height in nonathletic boys, consistent with previous studies in trained and untrained children.
10	Irawan (2017) [22]	16 players were involved in the analysis.	Plyometric group and Control group; Duration:- Plyometric group - 6 weeks, 3 times per week	Illinois agility test	The plyometric group increased their agility by 1.17 seconds after six weeks of training by focusing on stretch-shortening cycles to generate maximal power, stimulate proprioceptors, and improve intermuscular coordination by leveraging the muscle spindle and Golgi tendon organ.
11	Frohlich et al. (2014) [23]	There were 11 members included in the research.	Plyometric training group:- 2 times a week for 8 weeks	Squat jump, countermovement jump, drop jump, forehand overhead smashes	A plyometric training regimen of eight weeks can improve junior badminton athletes' jumping, hitting, and running skills, as well as their court speed and competitive ability. It should be personalized to individual needs, intensified with performance, and assessed with pre-, intermediate-, and post-tests.

Recommendations

Plyometric training can help badminton coaches, athletes, and trainers improve their agility and athleticism. To attain the best results, each player's particular strengths and weaknesses must be identified, and plyometric programs must be tailored accordingly. Regular and structured training is required, with an emphasis on establishing a solid foundation and gradually improving. Prioritizing form and technique can help to lessen the chance of injury. Plyometric training should be incorporated within a well-rounded training regimen that incorporates skill development, strength training, and aerobic conditioning. To minimize overtraining, coaches, athletes, and trainers must work together. Injury prevention strategies should be highlighted, such as enough rest periods, recovery focus, and monitoring for indicators of overuse or exhaustion. Communication and feedback are also essential. Coaches should keep open lines of contact with their athletes, understanding their development and altering their training program as appropriate. Athletes should provide comments on their plyometric performance both during and after the workout. Incorporating plyometric exercise into a badminton athlete's training program can boost performance, but it should be done with a strong emphasis on tailored training, moderate improvement, and a well-rounded training plan. Working together, coaches, athletes, and trainers can realize the full potential of plyometrics, ultimately boosting an athlete's agility, power, speed, and jumping ability, enhancing their competitive edge in badminton.

Designing Effective Plyometric Training Programs for Badminton

A structured strategy is required for plyometric training regimens for badminton players. A thorough assessment of the athlete's fitness level, strengths, limitations, and previous injuries is required before developing a specific training program. Agility exercises should improve court movement and reaction, while power and speed workouts should improve smashes and court coverage. Athletes should begin with fundamental exercises and gradually increase the intensity and complexity of their workouts over time. The program should be tailored to the athlete's overall training load, and rest intervals should be included to prevent overtraining. A comprehensive strategy incorporates skill development, strength training, and cardiovascular conditioning. It is critical for program efficacy and safety to monitor and make modifications depending on feedback. Finally, effective plyometric training programs for badminton players should be customized, progressive, and integrated into a larger training plan, with a focus on safety and injury prevention.

Safety Considerations and Injury Prevention Strategies

Badminton plyometric training necessitates a thorough approach that promotes safety and injury prevention. This entails assessing each athlete's fitness level, prior injuries, and training history in order to develop tailored routines. Proper technique and form are essential when practising plyometric activities, and coaches and trainers should provide regular feedback and correction. Gradual progression should start with simple workouts and progressively build intensity and complexity to ensure a firm foundation before moving on to more demanding routines. Plyometric training volume should be reasonable, with workout frequency and length matching the athlete's overall training load and allowing proper recuperation time. To increase recovery and reduce muscular discomfort, the warm-up should include dynamic stretching and mobility exercises, followed by a cool-down regimen. Rest intervals should be included between sets and exercises to avoid tiredness and damage. To avoid injuries during high-intensity activities, balance and stability training should be included. To avoid pushing athletes beyond their capabilities, adjustments and monitoring should be done. To reduce joint impact, plyometric training should be done on a soft surface with proper footwear. Cross-training can help to diversify sessions while also lowering the chance of overuse issues. Coaches, athletes, and trainers may ensure that plyometric training not only enhances agility and athletic proficiency but is also safe and responsible by adhering to safety considerations and injury prevention procedures. Maintaining athletes' health and well-being is critical for long-term success in badminton, which requires the capacity to move quickly and forcefully.

Discussion

Plyometric training has grown in popularity as an effective way to improve agility and athletic performance in a range of sports, including badminton. There has been a lot of interest in introducing plyometric training into badminton athletes' training routines in recent years since it offers a viable path for improving change of direction ability and performance growth. The goal of this review was to shed light on the potential benefits of plyometric training and its influence on badminton players for coaches, trainers, and athletes.

Maciejczyk et al. investigated the impact of short-term plyometric training on the agility, leap, and repeated sprint performance of female soccer players. The 17 participants were separated into two groups: PLY (plyometric) and CON (control). The PLY group improved significantly in leap and agility, but the CON group exhibited no significant changes. After four weeks, PLY was likewise effective [24]. Dass et al. investigated how anaerobic power and muscle strength were changed by strengthening and plyometric resistance training in badminton players. The study used an experimental design, with 40 individuals ranging in age from 18 to 24 divided into two groups: training and control. The training group showed a significant improvement in post-intervention levels, indicating that the training was effective in improving athletic performance [25]. In a study by Lee et al., after four weeks, supplemental physiotherapy or balance training programs had no effect on lower body power, balance, or reaction velocity in college basketball players. Possible causes include increased exposure, shorter training times, and already-trained participants. More research is needed to determine the impact of training intensity and movement requirements [26]. Guo et al. discovered that combining balance and plyometric training improved badminton athletes' change of direction (COD), meaning that combined training may be a more efficient COD training option for fitness trainers [27]. Buhril et al. used telemetric equipment and paired sample t-tests to examine the effect of plyometric exercise on the agility, speed, and anaerobic power of 20 male soccer players aged 18-27 [28]. Danardono et al. investigated how plyometric training influenced power and reactive agility in kumite karate competitors. The standing wide jump and standardized agility tests were used to collect data, and the treatment included four weeks of plyometrics modification exercises [29].

The purpose of the study carried out by Rasyid et al. was to see how a combination of plyometric and ladder drill training methods influences leg muscle speed, agility and power. The 36 male badminton players aged 12 to 16 were divided into three groups by the researchers: TR (tuck jump-double leg run), SH (squat jump-double leg hop), and C (conventional exercise). Each group received treatment three days each week for six weeks. Data was collected twice during the pretest and post-test. The multivariate analysis of variance (MANOVA) and post-hoc testing were used to assess the findings. Significant differences in speed, agility, and leg muscle power were found between the TR and SH groups, but not with the C group [30]. The Tennis-specific Agility Test (TAT) was developed and evaluated by 69 tennis players, showing moderate reliability, moderate concurrent validity, and high feasibility. The TAT captures physical and cognitive agility performance, making it a valuable tool for tennis players [31].

COD performance is critical in sports, and research has shown that eight weeks of plyometric training can increase it in soccer players. Drop jump (DJ) training for six weeks improved the results of the Illinois agility test, square test, Nebraska test, t-test, and hexagon test in DJ40cm, DJ60cm, and DJ80cm. DJ60cm had the highest effect size (ES), indicating that the DJ60cm intervention had the biggest impact on COD performance. The study also discovered that six weeks of DJ training at 40, 60, and 80 cm increased horizontal explosive performance in collegiate Sanda athletes, which aided COD performance. The ideal drop height for collegiate Sanda athletes to boost lower limb explosive and COD performance, however, was unknown, and more research on different drop heights was required [32].

A study was conducted by Chuang et al. on the effects of agility training on skill-related physical capabilities in young volleyball players. The goal was to examine the effects of different agility training methods on the physical capacities of adolescent volleyball players. There were 27 female players divided into three groups: shuttle-run training (STG), agility-ladder training (ATG), and control group (CG). The training session was held three times per week for six weeks. The participants' performances were evaluated both before and after the training. The results of the digging agility test (DAT) and agility T-test, and the 10-metre sprint (10MS) performance improved significantly in the STG and ATG groups. However, there were significant differences between the STG and CG groups in the DAT and agility T-test. The STG and ATG were shown to be significantly superior to the CG in the 10MS [33]. The study found that sports-specific agility training significantly improved tennis performance in male university-level players, with improved agility skills being transferable into sports-specific performance [34].

The goal of the study by Hotwani et al. was to determine how plyometrics and speed drills affected agility in lawn tennis players. Speed training enhanced agility, according to the study, with training focusing on footwork transition and switching from linear to lateral movement. Plyometric training, which includes jumping, drop jumps, counter-movement leaps, hopping, and other simple jumping exercises, significantly boosted agility over a three-week training program. Plyometric training improved power production, power yield, vertical bounce speed, hop stature, and muscle fibre size. Plyometric training improves individual performance and is more beneficial to tennis players than other conditioning approaches [35].

## Conclusions

Research on the association between plyometric training, agility, and athletic proficiency in badminton athletes discovered that it can improve their agility, power, speed, and jumping abilities significantly. Plyometric exercises like ladder exercises and shuttle runs improve court mobility and reactivity, whilst depth jumps and vertical leaps improve their ability to hit high shots and conduct powerful smashes. Plyometric exercises can assist athletes prepare for explosive court actions by incorporating them into warm-up routines. The study underlines the significance of personalized training, progressive exercise selection, and a well-rounded training regimen that combines skill development, strength training, and cardiovascular fitness. It also emphasizes the significance of injury prevention strategies such as balance and stability training, as well as the need for continuous monitoring and modifications to maximize the effectiveness of plyometric training. These findings imply that plyometric training can be a dynamic tool for enhancing agility and athletic ability, offering a competitive edge in the realm of badminton. These implications go beyond performance enhancement and underline the significance of holistic training that encompasses all aspects of a badminton athlete's development.
